# Local and introduced lineages drive MERS-CoV recombination in Egyptian camels

**DOI:** 10.1128/jvi.00641-25

**Published:** 2025-12-04

**Authors:** Mokhtar Gomaa, Kimberly M. Edwards, Ruixuan Wang, Ahmed El Taweel, Yassmin Moatasim, Omnia Kutkat, Mina Nabil Kamel, Hamdy A. El-Nagar, Vijaykrishna Dhanasekaran, Mohamed Ahmed Ali, Ghazi Kayali, Rabeh El-Shesheny

**Affiliations:** 1 Center of Scientific Excellence for Influenza Viruses, National Research Centre 68787, Giza, Egypt; 2 School of Public Health, LKS Faculty of Medicine, The University of Hong Kong 90397, Hong Kong SAR, China; 3 HKU-Pasteur Research Pole, LKS Faculty of Medicine, The University of Hong Kong 71075 , Hong Kong SAR, China; 4 Animal Production Research Institute, Agricultural Research Center 68888, Giza, Egypt; 5 Human Link DMCC, Dubai, United Arab Emirates; Emory University School of Medicine, Atlanta, Georgia, USA

**Keywords:** camels, MERS-CoV, zoonotic outbreaks, Egypt, seroprevalence, incidence

## Abstract

Dromedary camels are the primary reservoir for Middle East respiratory syndrome coronavirus (MERS-CoV), a zoonotic coronavirus responsible for sporadic human infections. While clade B predominates in the Arabian Peninsula and is frequently linked to zoonotic outbreaks and household secondary transmission, clade C circulates primarily in African camels, with limited evidence of human infections. The extent of MERS-CoV transmission, genetic diversity, and cross-species potential in North Africa remains poorly characterized. Here, we investigate MERS-CoV incidence, seroprevalence, and genomic recombination in dromedary camels and sympatric livestock across slaughterhouses and farms in Egypt. MERS-CoV was detected in 12% of camels sampled at slaughterhouses, with no evidence of infection in cattle, buffalo, sheep, or goats. Seroprevalence was higher in slaughtered camels (79%) than camels on farms (12%). Phylogenetic analyses of MERS-CoV genomes obtained from dromedary camels revealed an introduction of clade B into Egypt, originating from the Arabian Peninsula. Furthermore, we identified recombination events between clades B and C, in addition to events within each clade. This included at least one clade C virus that acquired multiple genomic regions from the newly introduced clade B viruses. These findings suggest that newly introduced MERS-CoV strains can recombine with locally circulating viruses, generating novel variants with potential zoonotic implications and challenging assumptions of limited cross-regional exchange. Enhanced surveillance, targeted control measures, and a One Health approach are crucial to mitigating MERS-CoV transmission and the emergence of recombinant strains.

**IMPORTANCE:**

This study highlights the importance of monitoring Middle East respiratory syndrome coronavirus (MERS-CoV) in dromedary camels, which are the main animal source of this virus that can occasionally infect humans. While most human cases have been linked to strains in the Arabian Peninsula, this research focused on Egypt, where the virus is less understood. Among surveyed dromedary camels and associated livestock, a significant number of camels at slaughterhouses were infected, and many had antibodies showing past exposure. Importantly, we discovered that a strain common in the Arabian Peninsula had recently entered Egypt and mixed genetically with local strains. This mixing, or recombination, can lead to new virus versions that may pose new risks to humans. The findings challenge the belief that MERS-CoV strains in different regions do not interact and highlight the need for stronger monitoring and prevention strategies. A One Health approach, linking animal, human, and environmental health, is key to managing future risks.

## INTRODUCTION

Middle East respiratory syndrome coronavirus (MERS-CoV) is a zoonotic betacoronavirus that originated in bats and is now endemic in dromedary camels, its primary reservoir (1). Zoonotic infection primarily occurs through direct or indirect contact with infected dromedary camels (2). Secondary human-to-human transmission has been reported among household members, healthcare workers, and other patients in close contact with infected individuals (3). Since its emergence in 2012, a total of 2,613 confirmed MERS-CoV cases have been reported globally, resulting in 943 deaths (case fatality rate ~36%, as of May 2024). These cases occurred across all WHO epidemiological regions in 27 countries. Saudi Arabia remains the epicenter, with 2,204 cases and 862 fatalities (39%), including four fatal cases reported in 2024 (4).

MERS-CoV has evolved into three distinct clades (A, B, and C) with clear geographic associations. Clade B has largely displaced clade A in the Arabian Peninsula and frequently causes zoonotic infections, whereas clade C circulates primarily among camels in Africa (5, 6). The zoonotic potential of MERS-CoV differs between clades B and C due to a combination of viral genetics and human behavior (7). Human seroprevalence rates vary depending on contact type, ranging from 0.15% in the general Saudi population to 2%–14% in high-risk groups such as farm workers, slaughterhouse workers, and camel racing workers (8, 9).

The dipeptidyl peptidase 4 (DPP4) receptor, the cellular entry point for MERS-CoV, is conserved across multiple mammalian species, raising concerns about interspecies transmission (10). MERS-CoV RNA and antibodies have been detected in animals with direct camel contact, including sheep, goats, cattle, and donkeys (11–13), but their role in viral maintenance is unclear. Understanding transmission dynamics, genetic diversity, and viral recombination in regions with frequent camel trade, such as North Africa, is essential for assessing zoonotic risk.

Here, we investigate MERS-CoV incidence, seroprevalence, and genomic diversity and recombination in dromedary camels and sympatric livestock across slaughterhouses and farms in Egypt. We assess the role of live animal markets in viral transmission, characterize the evolutionary relationships between local and introduced MERS-CoV lineages, and evaluate the potential for interspecies transmission. Our findings provide critical insights into MERS-CoV epidemiology in North Africa and inform strategies for surveillance and risk mitigation.

## MATERIALS AND METHODS

### Study design and sample collection

Between May 2023 and February 2024, nasal swabs (*n* = 270) and blood samples (*n* = 202) were collected from dromedary camels and co-housed livestock (cattle, water buffalo, sheep, and goats) to determine MERS-CoV incidence and seroprevalence. Samples were collected from live animals on farms in Marsa Matruh Governorate (northwestern coast) and from slaughtered animals at two abattoirs in Giza Governorate (Greater Cairo, central north). Camels and accompanying livestock were co-housed at both sampling sites. Swabs were placed on ice in viral transport medium (Dulbecco’s Modified Eagle Medium [DMEM]) supplemented with 4% fetal bovine serum and 5% antibiotic-antimycotic mix (Gibco, Life Technologies). Blood samples were drawn from the jugular vein using 20-gauge needles and vacuum serum gel tubes, centrifuged at 1,500 × *g* for 5 minutes, and sera were stored at −20°C.

### MERS-CoV detection

Nasal swabs were tested for MERS-CoV using WHO guidelines for laboratory testing (14). Viral nucleic acids were extracted with the MagMAX Viral/Pathogen Nucleic Acid Isolation Kit (Applied Biosystems) on a KingFisher Flex Purification System. RNA extracts (200 µL per nasal swab sample, 50 µL elution) were tested using real-time RT-PCR targeting upstream of the envelope gene (upE) (AgPath-ID One-Step RT-PCR Kit, Applied Biosystems). Positive samples underwent confirmatory *ORF1a* real-time RT-PCR (15).

### Microneutralization assay for anti-MERS-CoV antibodies

MERS-CoV MN assay using live virus was performed in a class III biosafety cabinet. Neutralizing antibodies were assessed using MERS-CoV/dromedary camel/Egypt/NC270 (16). Sera were heat-inactivated at 56°C for 30 minutes and serially diluted twofold starting at 1:10 in DMEM with 4% BSA and 1% antibiotic-antimycotic. Diluted sera were incubated with 100 TCID_50_ of the virus and incubated for 1 hour at 37°C. Vero-E6 cells in 96-well plates were inoculated with 50 µL per well and incubated for 90 minutes at 37°C. The inoculum was removed, the cells were supplemented with 150 µL DMEM per well, and plates were incubated for 3 days at 37°C with 5% CO_2_. The highest serum dilution inhibiting the viral cytopathic effect was recorded as the neutralizing titer, and animals with titers ≥20 were considered seropositive.

### Genome sequencing

cDNA synthesis was performed using the SuperScript IV First-Strand System (Invitrogen, MA, USA) with random hexamers. MERS-CoV genomes were amplified by multiplex PCR (Q5 High-Fidelity DNA Polymerase, New England Biolabs) using two in-house-designed primer pools (Table S1) to generate ~800 bp overlapping fragments. Amplicons were purified (GFX PCR DNA and Gel Band Purification Kit, GE Healthcare), and sequencing libraries were prepared using the Nextera XT DNA Library Prep Kit (Illumina). Libraries were sequenced on an Illumina MiniSeq with 150 bp paired-end reads. Sequence contigs were assembled using CLC Genomic Workbench (CLC Bio, Qiagen). Ten complete MERS-CoV genome sequences from this study were deposited into GenBank (Table S2).

### Phylogenetic analysis and recombination detection

MERS-CoV genomes from dromedary camels were analyzed alongside publicly available nucleotide sequences >20 kb in length with complete collection dates from 2018 to 2023 inclusive, downloaded from GenBank on 12 May 2025. Two to three sequences were selected from each transmission cluster (where sequence metadata indicated the same collection date and location), and identical sequences were removed. Accession numbers and strain names of sequences used for recombination analysis are provided in Table S3.

Sequences were aligned with MAFFT v.7.520 (17), and recombination was initially assessed with Recombination Detection Program 5 (RDP5) v.5.64 (18) using the following tools: RDP, GENECONV (for deep divergence), MaxChi (sensitive for small breakpoints), and 3SEQ (useful for recent events). Bonferroni correction was applied to reduce false positives. Breakpoints were further assessed with Genetic Algorithms for Recombination Detection (GARD) in HyPhy (19) under the GTR + Γ nucleotide substitution model, which uses phylogenetic incongruence and stepwise model selection to infer the number and location of breakpoints across the genome based on AICc. Recombination events involving the 2023 Egyptian camel strains were considered significant if corroborated by two or more detection methods.

To contextualize topology discordance, the nucleotide alignment was split at probable breakpoints, and maximum-likelihood trees were constructed using IQ-TREE2 (20), with 1,000 bootstrap replicates. SimPlot++ v.1.3 (21) was used to illustrate the inferred clade B and C recombination events. For each breakpoint-free region, temporal signal was confirmed via root-to-tip regression of genetic distance against sample collection date in TempEst v.1.5.3 (22). Time-scaled phylogenies were then constructed using TreeTime v.0.11.3 (23), and phylogenies with the best temporal signals (Fig. S1) were used to estimate the timing of recombination events.

## RESULTS

### Demographics of camels and sympatric livestock

A total of 270 animals were sampled to assess MERS-CoV prevalence and seroprevalence (Table 1). The majority of camels (*n* = 130; 81%) were sampled at slaughterhouses in Giza Governorate, while the remainder were sampled on farms in Matruh Governorate. Cattle, water buffalo, and 37% of sheep (*n* = 19/51) were sampled at slaughterhouses, while the remaining sheep (*n* = 32/51) and all goats were sampled on farms (Table 1).

**TABLE 1:** Sample collection and host demographics

	Dromedary camel	Cattle	Water buffalo	Sheep	Goat
Location, no. (%)					
Abattoir	130 (81)	36 (100)	6 (100)	19 (37)	0 (0)
Farm	34 (19)	0 (0)	0 (0)	32 (63)	13 (100)
Age, no.; median (range)^*a*^					
Adult	149; 10 (5–22)	36; 3 (2–5)	6; 3 (3–5)	51; 2 (1–3)	7; 3 (2–4)
Subadult	9; 4 (3–4)	0; 0 (0–0)	0; 0 (0–0)	0; 0 (0–0)	6; 1 (1–1)
Juvenile	6; 1.25 (1–2)	0; 0 (0–0)	0; 0 (0–0)	0; 0 (0–0)	0; 0 (0–0)
Sex, no. (%)					
Female	33 (20)	0 (0)	4 (67)	40 (78)	13 (100)
Male	131 (80)	36 (100)	2 (33)	11 (22)	0 (0)
Sampling visits, no. (%)					
May 2023	16 (9.75)	4 (11.11)	1 (16.67)	0 (0)	0 (0)
June 2023	71 (43.3)	22 (61.11)	5 (83.33)	19 (37.25)	0 (0)
July 2023	24 (14.63)	6 (16.67)	0 (0)	0 (0)	0 (0)
Nov 2023	19 (11.59)	4 (11.11)	0 (0)	0 (0)	0 (0)
Feb 2024	34 (20.73)	0 (0)	0 (0)	32 (62.75)	13 (100)
Total sampling	164 (100)	36 (100)	6 (100)	51 (100)	13 (100)

^*a*^ Ages are shown in years.

Most camels were adults (*n* = 149, median age = 10 years), with a small number of juveniles (*n* = 6, <2 years) and subadults (*n* = 9, 3–4 years). Accompanying animals included cattle (*n* = 36), water buffalo (*n* = 6), sheep (*n* = 51), and goats (*n* = 13) (Table 1). All cattle, water buffalo, and sheep were adults. Among goats, seven were adults (2–4 years) and six were subadults (~1 year). The majority of camels (80%) and all cattle were male, while most sheep (78%), water buffalo (67%), and all goats were female.

### MERS-CoV incidence and seroprevalence

During two sampling visits, 15 days apart in late 2023, 12.2% (*n* = 20/164) of dromedary camels sampled at slaughterhouses in the Giza Governorate tested positive for MERS-CoV. In November, 56.5% (13/23) of adult male camels tested positive, decreasing to 29.2% (7/24) in December. MERS-CoV was not detected from the nasal swabs of camels sampled on farms, and the nasal swabs of all co-housed livestock also tested negative by real-time RT-PCR.

Overall, 60% of camels had neutralizing antibodies to MERS-CoV (titers ≥20). Seroprevalence was significantly lower in camels on farms (*n* = 4/34; 12%) than in slaughterhouse camels (79%; *P* < 0.0001) (Table 2). Among accompanying animals, 17% (*n* = 4/24) of bulls at the slaughterhouse were seropositive, including three with titers of 20 and one with a titer of 160. Two sheep at the slaughterhouse also tested seropositive (titers: 20, 80), while all five water buffaloes were seronegative. All sheep and goats sampled on farms in Matruh Governorate were seronegative for MERS-CoV antibodies (Table 2).

**TABLE 2:** MERS-CoV detection from slaughterhouses and farms

Livestock species	PCR-positive nasal swab	Seropositivity
Abattoir		
Dromedary camel	20/130 (15%)	67/85 (79%)
Water buffalo	0/6 (0%)	0/5 (0%)
Cattle	0/36 (0%)	4/24 (17%)
Sheep	0/19 (0%)	2/9 (22%)
Goats	0/0 (0%)	0/0 (0%)
Farm		
Dromedary camel	0/34 (0%)	4/34 (12%)
Water buffalo	0/0 (0%)	0/0 (0%)
Cattle	0/0 (0%)	0/0 (0%)
Sheep	0/32 (0%)	0/32 (0%)
Goats	0/13 (0%)	0/13 (0%)

### MERS-CoV recombination in dromedary camels

Ten MERS-CoV viruses were sequenced from dromedary camel nasal swabs (Table S2). An initial maximum-likelihood phylogeny indicated that 9 of the 10 camel isolates clustered with West African clade C camel strains, while MERS-CoV/dromedary camel/Egypt/STM0244/2023 (STM0244) clustered with clade B strains, suggesting an introduction of clade B into Egypt.

Although only a partial genome sequence could be recovered from the recombinant Egyptian camel isolate MERS-CoV/dromedary camel/Egypt/STM0184/2023 (STM0184), RDP5 tools identified multiple portions of its genome that were acquired from the newly introduced clade B strain, STM0244, including portions of *ORF1ab*, spike, and nucleocapsid proteins (Table 3; Fig. S2). Additional clade B and C recombination events were detected in the spike of STM0184 and *ORF1ab* of strain STM0186, but with insufficient statistical support (Table S4). Egyptian camel strains STM0185, STM0186, STM0189, and STM0199 showed evidence of historical recombination within clade C, but these events also lacked sufficient statistical backing (Table S4).

**TABLE 3:** Recombination events detected in Egyptian camel strains^*b*^

Position in alignment (95% CI)		Accession no. (Egyptian camel isolate)			RDP5 detection method (*P*-value)			
Start	End	Recombinant	Minor parent	Major parent	RDP	GENECONV	MaxChi	3SEQ
Clade B/C								
12534 (11282–13421)	13618 (13594–14836)	PV239404 (STM0184)	PV239413 (STM0244)	PV239411 (STM0199)	4.60E − 08	3.06E − 06	1.80E − 05	4.17E − 04
16515^*a*^ (15973–18031)	19245^*a*^ (18134–28355)	PV239404 (STM0184)	PV239413 (STM0244)	MK462247	1.63E − 05	2.32E − 04	NS	2.56E − 02
29428^*a*^ (29156–29746)	30022 (undetermined)	PV239404 (STM0184)	PV239413 (STM0244)	PV239411 (STM0199)	NS	2.13E − 02	1.46E − 02	NS
Clade B								
17910^*a*^ (16630–17941)	24468^*a*^ (22978–25186)	PP952163	PV239413 (STM0244)	PP952170	3.83E − 06	1.57E − 03	5.37E − 07	6.85E − 07

^*a*^ Breakpoint estimate corroborated by GARD.

^*b*^ NS, non-significant *P*-value (>0.05) for recombination event.

**Fig 1:**
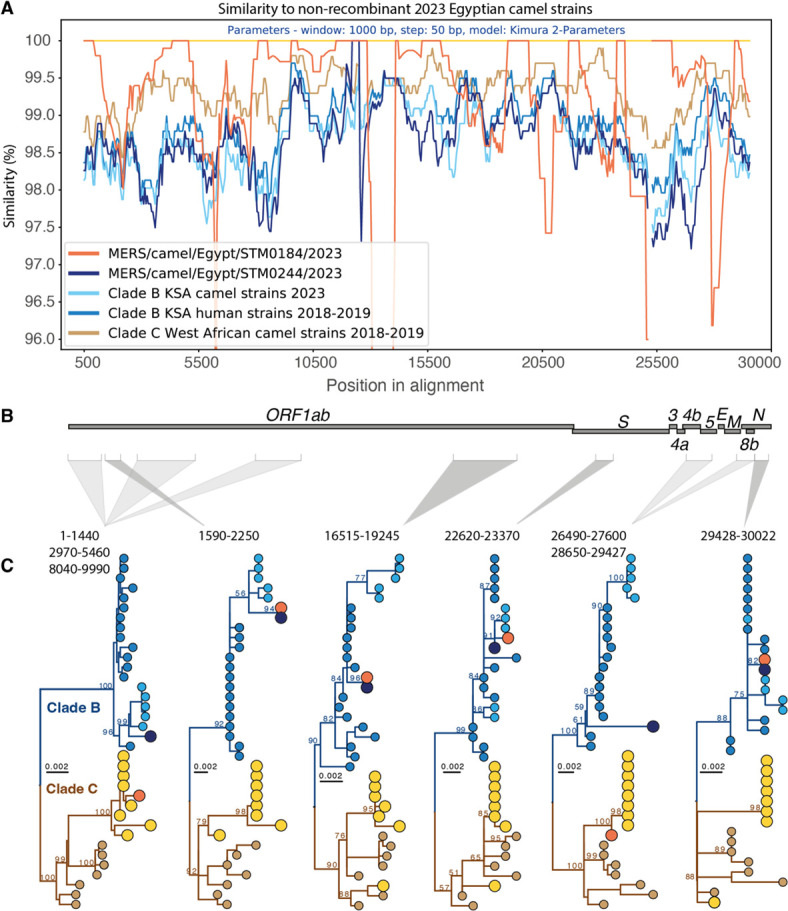
MERS-CoV recombination in Egyptian dromedary camels. (**A**) Nucleotide similarity to non-recombinant 2023 Egyptian camel strains (PV239408, PV239409, PV239410, PV239412) identified with SimPlot++, using the Kimura-2 parameter model, a 1,000 bp window, and 50 bp step. (**B**) Genomic regions selected for phylogenetic inference, accounting for breakpoint confidence intervals and gaps in the recombinant strain, MERS-CoV/dromedary camel/Egypt/STM0184/2023. (**C**) Maximum-likelihood trees from selected contigs. Bootstrap values (1,000 replicates) are shown on key nodes; scale bars indicate nucleotide substitutions per site. Tip colors correspond to panel **A**. Accession numbers are provided in Tables S2 and S3. To examine topological discordance and infer the evolutionary history of the interclade recombination, genomic regions were selected based on the breakpoint estimates and their confidence intervals, while accounting for gaps in the recombinant sequence, STM0184 (Fig. S2). Blocks with the strongest temporal signals were used to infer timing of recombination events (Fig. S1). Within recombinant regions of the *ORF1b* (nucleotide positions 16515–19245) and nucleoprotein (24948–30022), STM0244 and STM0184 sequences shared 100% sequence homology (panel C). Based on the *ORF1b* contig, this recombination event most likely occurred around late October 2023 (90% credible interval [CI]: March–November 2023) (Fig. 2A).

GARD, which identifies recombination based on phylogenetic incongruence, detected significant phylogenetic recombination in the 36-sequence MERS-CoV alignment (30,051 sites). The best-fitting model contained nine breakpoints, at positions 4090, 6241, 8314, 10627, 16570, 17910, 21684, 24470, 27278, and 29427. There was strong evidence for recombination (ΔAICc = 35.73 compared to the single-tree model), indicating a significantly better fit with recombination. To visualize recombination patterns across the genome, the sequence similarity was then compared against clade B and C reference groups using SimPlot++ (Fig. 1A).

**Fig 2:**
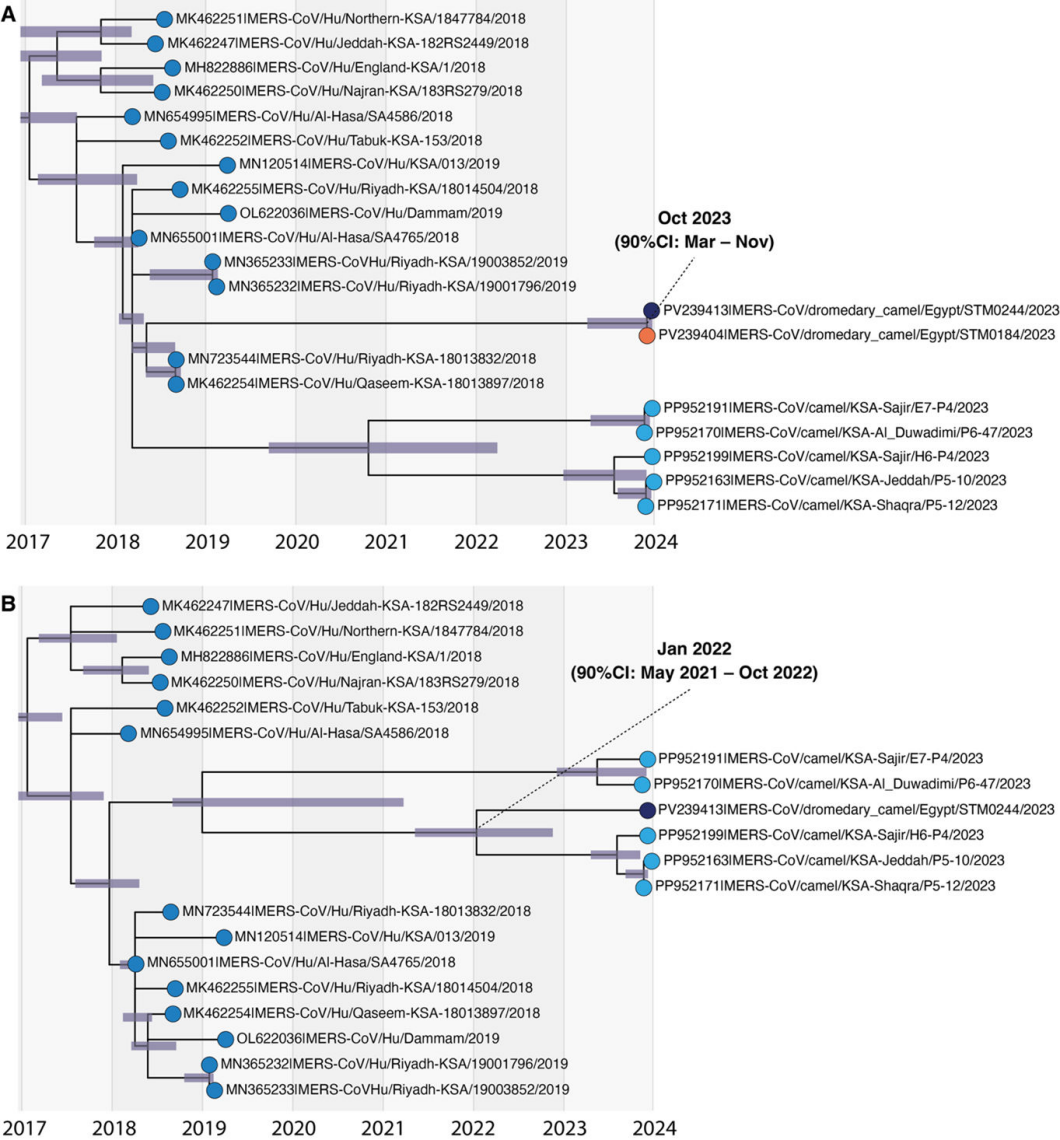
Inferred timing of clade B recombination events. Time-resolved maximum-likelihood phylogenies with 90% credible intervals (shaded bars) representing inferred node dates. (**A**) Estimated timing of recombination between strains STM0244 (dark blue) and STM0184 (orange), based on nucleotide positions 16515–19245. (**B**) Estimated timing of clade B recombination involving STM0244 and 2023 Saudi Arabian camel strains (light blue), based on nucleotide positions 17910–24468.

Across nearly all of the genomic regions analyzed, the clade B isolate, STM0244, shared common ancestry with Saudi Arabian camel strains isolated in November and December of 2023 (Fig. 1C). However, a historical recombination among clade B strains was detected around positions 17910–24468, presumably involving a strain ancestral to STM0244 and the 2023 Saudi Arabian camel isolates (Table 3). Time-measured phylogenetic estimates suggest the clade B recombination occurred around January 2022 (90% CI: May 2021–October 2022) (Fig. 2B).

## DISCUSSION

This study provides critical insights into the prevalence, seroprevalence, and evolutionary dynamics of MERS-CoV among dromedary camels and associated livestock in Egypt. Consistent with previous studies identifying dromedary camels as the primary MERS-CoV reservoir, our findings revealed significantly higher viral prevalence in camels sampled at slaughterhouses compared to farms. Besides the fact that anti-coronavirus antibodies wane over time (24, 25), the sampled farm implemented a quarantine policy for adding new animals. On the other hand, slaughterhouses usually aggregate animals from diverse geographic origins within Egypt and those imported from other countries, likely serving as hubs for viral mixing and amplification and increasing the potential for zoonotic spillover (26–31). This pattern parallels findings from other livestock-associated viruses, such as H7N9 influenza virus, where trade hubs and live markets have served as critical nodes for viral dissemination (32). In contrast, the absence of MERS-CoV among farmed camels and accompanying livestock highlights the protective effect of controlled environments and restricted animal movement. These results emphasize the role of slaughterhouses and live animal markets in amplifying MERS-CoV transmission along the supply chain, reinforcing the importance of biosecurity measures to limit inter-herd contact and viral spread. It is important to note that a very small number of non-camelid mammals were included in this study, and the results may not reflect the true incidence or prevalence of MERS-CoV.

Genomic analyses identified multiple recombination events, both within and among clades B and C, reflecting the well-documented role of recombination in coronavirus evolution (33). Recombination can generate viral variants with altered phenotypic traits, as seen in the emergence of MERS-CoV, in which the ancestral receptor binding domain was replaced by that of another merbecovirus lineage, modifying receptor usage (34). The identification of recombinant MERS-CoV strains in this study suggests that international camel trade is a key driver of viral diversity. Given the potential for recombination to produce strains with increased zoonotic potential, sustained regional and international surveillance is essential to monitor evolutionary trends and emerging risks.

The precise timing and breakpoint positions of these recombination events remain uncertain due to the limited number of contemporary MERS-CoV sequences, missing bases in the sequenced genomes, and frequency of coronavirus recombination. Additionally, there was some discordance among the tools used to infer breakpoints, attributable to differences in their underlying algorithms, sensitivity, and specificity. Genetic distance-based methods (i.e., 3SEQ and RDP) have a high false-positive rate and are more sensitive to recent, minor recombination events, whereas tools that infer recombination based on topological discordance, such as GARD, are better suited to examine deep discordance and interclade recombination. We therefore only considered breakpoints that were corroborated by strong statistical support from multiple detection tools.

The dissemination of potentially zoonotic clade B viruses into North Africa and interclade recombination is cause for concern. Time-scaled phylogenetic analyses suggest that the clade B and C recombinant may have circulated undetected for several months. These findings highlight the urgent need for sustained surveillance in animals to better understand MERS-CoV evolutionary dynamics and the zoonotic potential of emerging strains. Although MERS-CoV elimination remains unfeasible due to its endemicity in dromedary camels and logistical barriers to large-scale interventions, targeted control strategies offer pathways to mitigate future zoonotic spillover. A One Health framework—integrating veterinary, human, and environmental health surveillance—will be crucial for managing MERS-CoV risks. Key strategies include enhanced biosecurity in high-risk settings and public awareness campaigns focused on reducing human-animal contact.

In conclusion, this study underscores the interconnected roles of viral evolution, international trade, and supply chain dynamics in shaping MERS-CoV epidemiology. The observed recombination events and prevalence patterns reinforce the need for coordinated, regional surveillance to detect emerging variants and mitigate public health risks. Strengthened biosecurity measures, targeted interventions, and sustained One Health efforts will be essential to minimize MERS-CoV transmission and reduce the risk of future outbreaks.

## Data Availability

Sequences from this study were deposited in GenBank under accession numbers PV239404, PV239405, PV239406, PV239407, PV239408, PV239409, PV239410, PV239411, PV239412, and PV239413.

## SUPPLEMENTAL MATERIAL

Supplemental material jvi.00641-25-s0001.docx Figures S1 and S2; Tables S1 to S4.

ASM does not own the copyrights to Supplemental Material that may be linked to, or accessed through, an article. The authors have granted ASM a non-exclusive, world-wide license to publish the Supplemental Material files. Please contact the corresponding author directly for reuse.
