# Supplementary material for: Local and introduced lineages drive MERS-CoV recombination in Egyptian camels
Source: J Virol. 2025 Dec 4;99(12):e00641-25. doi: 10.1128/jvi.00641-25 (PMC12724290; doi:10.1128/jvi.00641-25)
Supplement: Supplemental material — Figures S1 and S2; Tables S1 to S4. [file jvi.00641-25-s0001.docx]

**
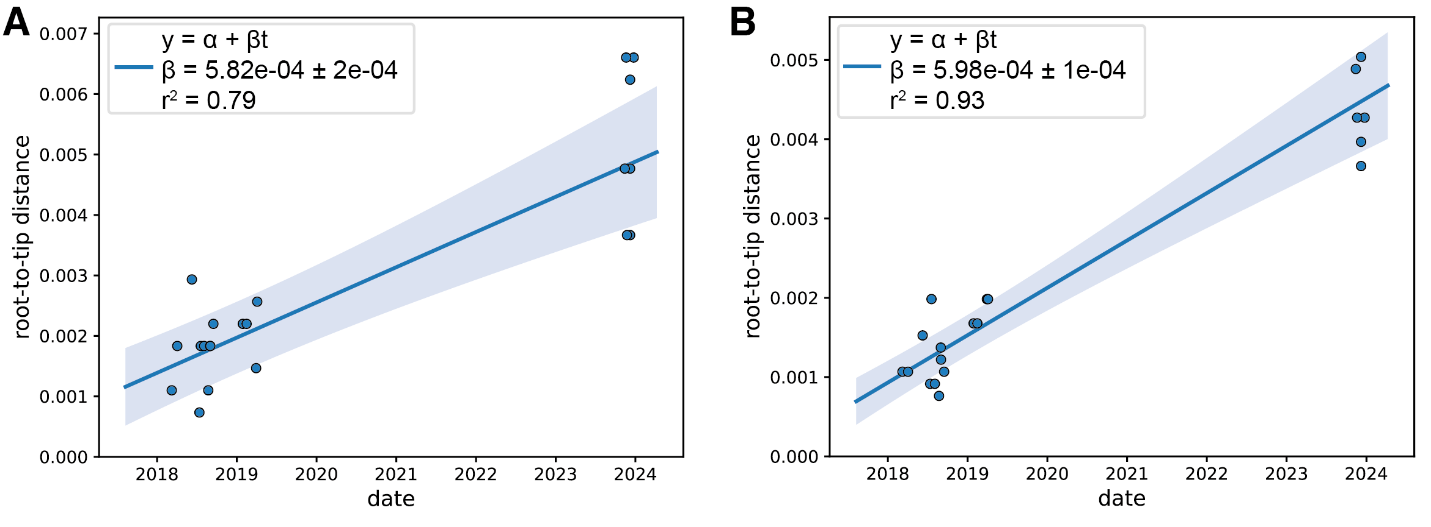
**

**Figure S1. Temporal signal in genomic blocks selected to infer timing of recombination events.** Regression of root-to-tip genetic distance and sample collection date, based on maximum likelihood tree reconstructions with a root position that maximizes the residual mean squared (shown in Figure 2). (A) Nucleotide positions 16,515–19,245, used to estimate timing of recombination between strains STM0244 (GenBank accession no. PV239413) and STM0184 (PV239404). (B) Nucleotide positions 17,910 – 24,468, used to infer timing of clade B recombination involving STM0244 and 2023 Saudi Arabian camel strains.

**
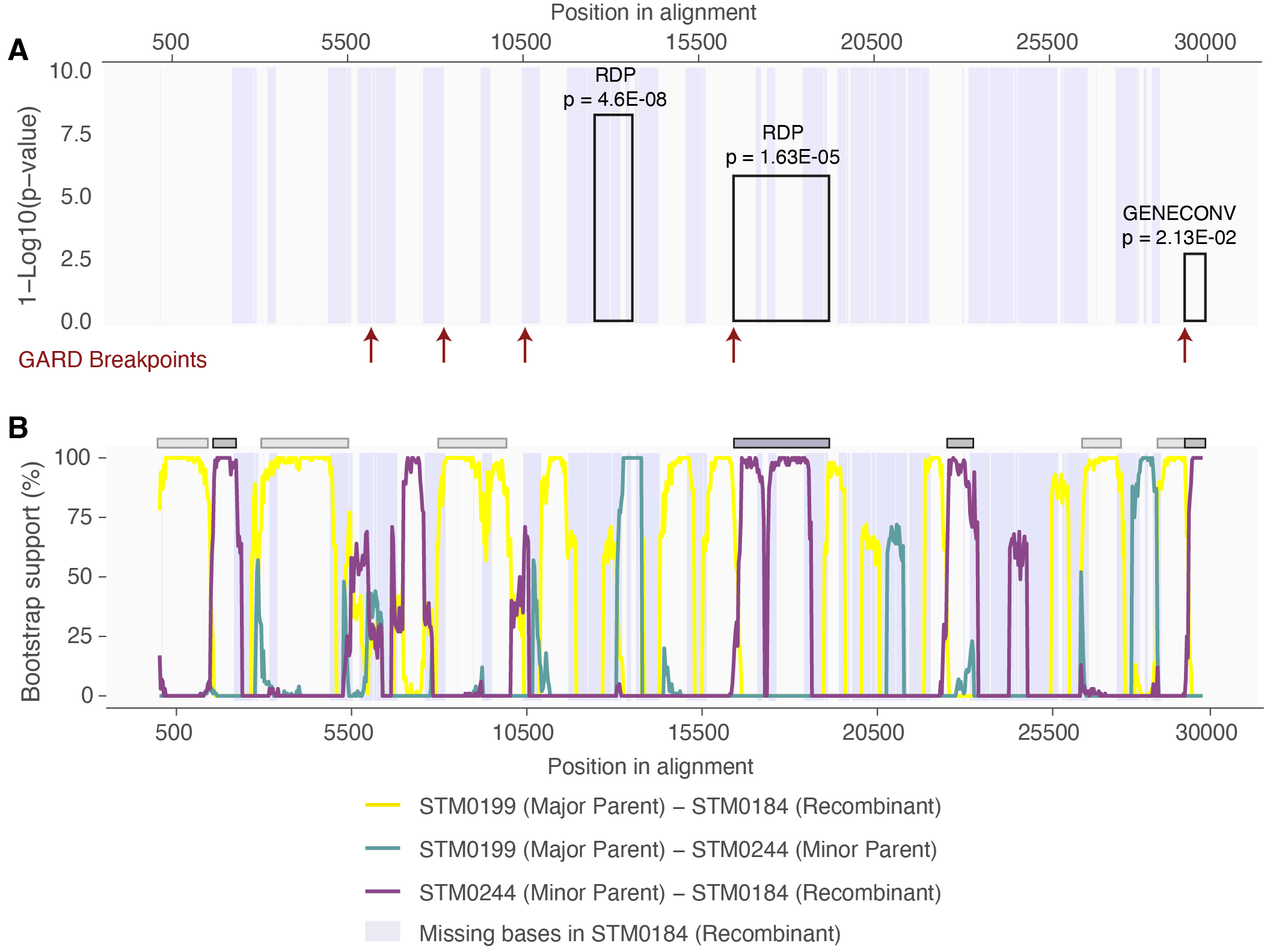
**

**Figure S2. Statistical support for recombination between clades.** Purple shading indicates gaps in the aligned recombinant sequence, MERS-CoV/dromedary camel/STM0184/2023 (PV239404). (A) Best p-value and method of detection for recombination events with MERS-CoV/dromedary camel/STM0184/2023 (PV239404) as the recombinant strain and MERS-CoV/dromedary camel/Egypt/STM0244/2023 (PV239413) as the minor parental strain. Red arrows show the position of GARD breakpoints associated with these clade B and C recombination events. (B) Bootstrap support for potential recombination, estimated using the Kimura-2 model with a 500 bp window and 30 bp step size. Bars indicate non-recombinant (light gray) and recombinant (dark gray) blocks selected for phylogenetic inference.

**Table S1. MERS-CoV primers**

| Name | Sequence |
| --- | --- |
| MERS_1_LEFT | TCTCACTTCCCCTCGTTCTCTT |
| MERS_1_RIGHT | GCCTCAAAATCGTCCATCCACT |
| MERS_2_LEFT | TATTCTCCTGCGCAAGTATGGC |
| MERS_2_RIGHT | ACCAACTGAGCAACTGAATGTTTG |
| MERS_3_LEFT | GTCGAAGTCCAATCATCTGGCA |
| MERS_3_RIGHT | GCCAGTCTTATCTTGCAAGATGC |
| MERS_4_LEFT | ACATGGATTCTGGTGTGTCATCC |
| MERS_4_RIGHT | TGTACTTGATCACCGCCAAAGG |
| MERS_5_LEFT | GGATTTGCCTTCTACCCTGCTT |
| MERS_5_RIGHT | TGTCTGTTCGGTATCAGCCTCA |
| MERS_6_LEFT | GGAAACTCCTGTTGTGCCTGAT |
| MERS_6_RIGHT | CTTCTTAGTATAATCAACACCCTTGTTCC |
| MERS_7_LEFT | TCATATGATGGGTTACGTGGCG |
| MERS_7_RIGHT | CGCCTTTGTTTCATCAGCAGTC |
| MERS_8_LEFT | AGATCACAGCTTGGATGCGTTT |
| MERS_8_RIGHT | AAATAAGACCACCCTTCAGGCG |
| MERS_9_LEFT | CTGCTCTCAGGCACACCAAAT |
| MERS_9_RIGHT | CAGCAACGAAACTGACATTGTCC |
| MERS_10_LEFT | GTGGAGTCTACACCAGTTGAACC |
| MERS_10_RIGHT | CAAGACCGTCACAAGCAGAAGA |
| MERS_11_LEFT | TCACTTGTATGTCTTCAATCAGGTCTT |
| MERS_11_RIGHT | TGTGATCTATCCGTAGCGTTAATAGG |
| MERS_12_LEFT | TGTGTGGATTGTGACACTGCAG |
| MERS_12_RIGHT | CGTTCCAAATGCAAGCACCATT |
| MERS_13_LEFT | CCTGATAGTGTGTCTACCAGCG |
| MERS_13_RIGHT | GTAGGATCATGGCAGTATGGTGTC |
| MERS_14_LEFT | GCTACACTCCTATAGATGAGATACCCT |
| MERS_14_RIGHT | AAGCTAAAACCCAGAAGAAGTGTCT |
| MERS_15_LEFT | GCTACATTCTATTTTACTAATGAGCCTGC |
| MERS_15_RIGHT | GCTAACACACTAAATGCTGCGC |
| MERS_16_LEFT | CCATGCAAGGCACTCTTTTGAAG |
| MERS_16_RIGHT | GGGAACAACTGTGTGGGAATAGT |
| MERS_17_LEFT | GGTACTGCGCATTGGTTGTTTG |
| MERS_17_RIGHT | AGAGCCATAGCCTCCCAAGAAT |
| MERS_18_LEFT | CCTTAAGCTTAGAGCACCTATGGG |
| MERS_18_RIGHT | TTGAAGCACACAGTGGGATGAC |
| MERS_19_LEFT | TTGTCAGTGCTATGCAAACTATGTTG |
| MERS_19_RIGHT | AACACAATTTGTCAATGGGGCAC |
| MERS_20_LEFT | GGTTCTAACACCGAGTTTGCCT |
| MERS_20_RIGHT | CCTCAGGGCATATACAAGATCCATC |
| MERS_21_LEFT | TGCTGTAGCTCCCCATGATTTC |
| MERS_21_RIGHT | TGGAGACTAACATCCATATTCATGACT |
| MERS_22_LEFT | TGCCTAAGACTTGTTTCGGACC |
| MERS_22_RIGHT | CGAGTTGCAGCCATGGACTTAA |
| MERS_23_LEFT | GCTAAGAATAGAGCTCGCACTGT |
| MERS_23_RIGHT | GGCCCTTTCTTCAGATCGGTTT |
| MERS_24_LEFT | CAGCTAAGGGTTACATTGCTGGA |
| MERS_24_RIGHT | GGACTACCTGTGCACATATTCTTGT |
| MERS_25_LEFT | CCTGGTTGTGGCGTTTCAGA |
| MERS_25_RIGHT | CACACAAAGCATCAACAGCTGC |
| MERS_26_LEFT | CTGGCACTGGCAAAAGTCATTTT |
| MERS_26_RIGHT | TGGTATTCTGAACCCTGTGAGGA |
| MERS_27_LEFT | GGCATGGAGTAAGGCAGTCTTT |
| MERS_27_RIGHT | GACAACATTTGCACTATACGTCGTC |
| MERS_28_LEFT | GGGGTAACATGTTAACGGGCAT |
| MERS_28_RIGHT | CACGTGTGTCAAACCTGCATAC |
| MERS_29_LEFT | GGACATGGCCTCAAGATTTGCT |
| MERS_29_RIGHT | ACTTACATACACCAATAGTAGCAGTACC |
| MERS_30_LEFT | ACTCTATGCTAAGCGTGCTGTAC |
| MERS_30_RIGHT | TGAACCTGTCCATCCTTACACCA |
| MERS_31_LEFT | GCTTTTAAGGCGGTGTGTTCTG |
| MERS_31_RIGHT | ACCTAAGAGGAATCCTTCAGATGAG |
| MERS_32_LEFT | CAGAACACTCTTGGAGCGTTGA |
| MERS_32_RIGHT | GAAGAACCCAGCATAAAAGCAGG |
| MERS_33_LEFT | GCTAATGGGTTTGTCGTCCGTA |
| MERS_33_RIGHT | CTTCAGCCTGTTCCACAACTGA |
| MERS_34_LEFT | GACTGTGGTTTTAATGATTTGTCACAAC |
| MERS_34_RIGHT | CCCGAAACACCATAGAGGGAAT |
| MERS_35_LEFT | CAGATGGGCTTTGGTATTACAGTTC |
| MERS_35_RIGHT | CACCATGGAGAGCCTGGTTTATT |
| MERS_36_LEFT | GGAGTACATTCAGACAACCATTCAGA |
| MERS_36_RIGHT | CTGTCTATTTGGGCGTCCTGTT |
| MERS_37_LEFT | CAACAATGCACAGGCTCTATCCA |
| MERS_37_RIGHT | GAAACCAAGCCAAATGTACCACG |
| MERS_38_LEFT | CGATCTTACCTACGAGATGTTGTCT |
| MERS_38_RIGHT | CCTGTTTGGCCAAAGCTTTTGT |
| MERS_39_LEFT | TAGTTGGCACTTCATTGCACCC |
| MERS_39_RIGHT | AGACAACATCTACGGCCAAAGG |
| MERS_40_LEFT | GCACTGGATGATGTTACTCGCA |
| MERS_40_RIGHT | CAGGCACGAAAACAGTGGAAAC |
| MERS_41_LEFT | GCTGTTCTCGTTGTTTTTATTTGCAC |
| MERS_41_RIGHT | TAAACAGCCGGATACTCTGCAC |
| MERS_42_LEFT | CCATCTTCCATGGCGCTATCAA |
| MERS_42_RIGHT | GCCAGTTGCTTAATTCCATTCCC |
| MERS_43_LEFT | AAGTCCCTCTTACCTTTCCACCT |
| MERS_43_RIGHT | CACAGGGTTGCCATGATCATCA |
| MERS_44_LEFT | AAGAAAGATGCTGCTGCTGCT |
| MERS_44_RIGHT | TCACACTGTAGAGCTCTTCCCG |

**Table S2. GenBank accession numbers of MERS-CoV genomes isolated in this study.**

| Virus Isolate | Accession Number |
| --- | --- |
| MERS-CoV/dromedary camel/Egypt/STM0184/2023 | PV239404 |
| MERS-CoV/dromedary camel/Egypt/STM0185/2023 | PV239405 |
| MERS-CoV/dromedary camel/Egypt/STM0186/2023 | PV239406 |
| MERS-CoV/dromedary camel/Egypt/STM0189/2023 | PV239407 |
| MERS-CoV/dromedary camel/Egypt/STM0190/2023 | PV239408 |
| MERS-CoV/dromedary camel/Egypt/STM0191/2023 | PV239409 |
| MERS-CoV/dromedary camel/Egypt/STM0198/2023 | PV239410 |
| MERS-CoV/dromedary camel/Egypt/STM0199/2023 | PV239411 |
| MERS-CoV/dromedary camel/Egypt/STM0200/2023 | PV239412 |
| MERS-CoV/dromedary camel/Egypt/STM0244/2023 | PV239413 |

**Table S3. MERS-CoV sequences used to infer recombination events.**

| **GenBank**  **Accession** | **Virus name*** | **Country** | **Collection date** | **Clade** |
| --- | --- | --- | --- | --- |
| MH734114 | MERS-CoV/camel/Kenya/C1215/2018 | Kenya | 2018-03-13 | C |
| MH822886 | MERS-CoV/hu/England-via-KSA/1/2018 | Saudi Arabia | 2018-08-22 | B |
| MK462247 | MERS-CoV/hu/Jeddah-KSA/182RS2449/2018 | Saudi Arabia | 2018-06-08 | B |
| MK462250 | MERS-CoV/hu/Najran-KSA/183RS279/2018 | Saudi Arabia | 2018-07-12 | B |
| MK462251 | MERS-CoV/hu/Northern-KSA/1847784/2018 | Saudi Arabia | 2018-07-18 | B |
| MK462252 | MERS-CoV/hu/Tabuk-KSA/153/2018 | Saudi Arabia | 2018-08-03 | B |
| MK462254 | MERS-CoV/hu/Qaseem-KSA/18013897/2018 | Saudi Arabia | 2018-08-31 | B |
| MK462255 | MERS-CoV/hu/Riyadh-KSA/18014504/2018 | Saudi Arabia | 2018-09-15 | B |
| MN120514 | MERS-CoV/hu/KSA/013/2019 | Saudi Arabia | 2019-03-28 | B |
| MN365232 | MERS-CoV/hu/Riyadh-KSA/19001796/2019 | Saudi Arabia | 2019-01-28 | B |
| MN365233 | MERS-CoV/hu/Riyadh-KSA/19003852/2019 | Saudi Arabia | 2019-02-15 | B |
| MN654995 | MERS-CoV/camel/Al-Hasa/SA4586/2018 | Saudi Arabia | 2018-03-07 | B |
| MN655001 | MERS-CoV/camel/Al-Hasa/SA4765/2018 | Saudi Arabia | 2018-04-02 | B |
| MN723544 | MERS-CoV/hu/Riyadh-KSA/18013832/2018 | Saudi Arabia | 2018-08-30 | B |
| MZ268405 | MERS-CoV/camel/Kenya/HKU-CAC10200/2020 | Kenya | 2020-03-19 | C |
| OK094448 | MERS-CoV/camel/Kenya/M23C07/2019 | Kenya | 2019-04-30 | C |
| OL622036 | MERS-CoV/hu/Dammam/1/2019 | Saudi Arabia | 2019-04-03 | B |
| OP866286 | MERS-CoV/camel/Ethiopia-Akaki/CAC9650/2019 | Ethiopia | 2019-09-23 | C |
| OP866287 | MERS-CoV/camel/Ethiopia-Akaki/CAC9670/2019 | Ethiopia | 2019-10-29 | C |
| OP866288 | MERS-CoV/camel/Ethiopia-Akaki/CAC9691/2019 | Ethiopia | 2019-11-24 | C |
| OP866294 | MERS-CoV/camel/Ethiopia-Amibara/CAC11202/2019 | Ethiopia | 2019-11-23 | C |
| PP952163 | MERS-CoV/camel/KSA-Jeddah/P5-10/2023 | Saudi Arabia | 2023-12-23 | B |
| PP952170 | MERS-CoV/camel/KSA-Al_Duwadimi/P6-47/2023 | Saudi Arabia | 2023-11-13 | B |
| PP952171 | MERS-CoV/camel/KSA-Shaqra/P5-12/2023 | Saudi Arabia | 2023-11-19 | B |
| PP952191 | MERS-CoV/camel/KSA-Sajir/E7-P4/2023 | Saudi Arabia | 2023-12-07 | B |
| PP952199 | MERS-CoV/camel/KSA-Sajir/H6-P4/2023 | Saudi Arabia | 2023-12-07 | B |

*****Virus names are shown as “MERS-CoV/host/location/isolate_number/year”; hu, human; KSA, Kingdom of Saudi Arabia.

**Table S4. Additional recombination events with insufficient statistical support.**

|  | **Position in alignment** | |  | | | **RDP5 Detection Methods (p-value)** | | | |
| --- | --- | --- | --- | --- | --- | --- | --- | --- | --- |
|  | **Start** | **End** | **Recombinant** | **Minor Parent** | **Major Parent** | **RDP** | **GENECONV** | **Maxchi** | **3Seq** |
| **Clade B/C** |  |  |  |  |  |  |  |  |  |
|  | 219* | 1,560* | ^ OL622036 | † PV239411 (STM0199) | PV239404 (STM0184) | NS | NS | NS | NS |
|  | 10,521 | 21,820 | ^ PV239406 (STM0186) | † PP952170 | PV239409 (STM0191) | NS | NS | NS | NS |
|  | 22,529* | 25,803 | PV239404 (STM0184) | PV239413 (STM0244) | OK094448 | NS | NS | NS | NS |
|  | 28,226 | 28,582 | ^ PV239404 (STM0184) | † PP952199 | PV239411 (STM0199) | NS | NS | NS | NS |
| **Clade C** |  |  |  |  |  |  |  |  |  |
|  | 1,110* | 18,332 | ^ PV239405 (STM0185) | † OP866294 | PV239407 (STM0189) | NS | NS | NS | NS |
|  | 1,454* | 6,581 | ^ PV239407 (STM0189) | † OP866288 | PV239409 (STM0191) | NS | NS | NS | NS |
|  | 18,304* | 21,536 | PV239411 (STM0199) | † PV239410 (STM0198) | PV239407 (STM0189) | NS | NS | NS | NS |
|  | 24,305 | 30,050* | ^ PV239406 (STM0186) | PV239407 (STM0189) | † OP866286 | NS | NS | NS | NS |
|  | 24,855 | 25,208 | ^ PV239407 (STM0189) | † OP866286 | PV239409 (STM0191) | NS | NS | NS | NS |
| **Clade B** |  |  |  |  |  |  |  |  |  |
|  | 1,182* | 12,908 | ^ MK483839 | † PP952191 | PP952170 | NS | NS | NS | NS |

*Actual breakpoint position is undetermined (likely either overprinted by a subsequent recombination event or off the edges of the analysed sequence fragments).

^ Recombinant sequence may have been misidentified (one of the identified parents might be the recombinant).

† Sequence used to infer the existence of a missing parental sequence.

NS, Non-significant p-value (>0.05) for recombination event.
